# Maturation and Conversion of Somatic Embryos Derived from Seeds of Olive (*Olea europaea* L.) cv. Dahbia: Occurrence of Secondary Embryogenesis and Adventitious Bud Formation

**DOI:** 10.3390/plants9111489

**Published:** 2020-11-04

**Authors:** Mouaad Amine Mazri, Rachida Naciri, Ilham Belkoura

**Affiliations:** 1 Laboratoire de Biotechnologie Végétale, CRRA-Marrakech, UR Agro-Biotechnologie, Institut National de la Recherche Agronomique, Marrakech BP 533, Morocco; 2 Laboratoire de Culture In Vitro, Département des Sciences de Base, Ecole Nationale d’Agriculture, Meknes BP S/40, Morocco; nacirirachida@gmail.com (R.N.); bilham@enameknes.ac.ma (I.B.)

**Keywords:** adventitious bud, in vitro tissue culture, secondary embryogenesis, somatic embryogenesis

## Abstract

Maturation and conversion of somatic embryos are two crucial steps that hamper the development of efficient somatic embryogenesis systems in olive. Herein, a simple and efficient protocol for the maturation and conversion of olive somatic embryos is reported. Globular somatic embryos derived from seeds of cv. Dahbia were cultured on either half-strength olive (OM) or olive cyclic embryogenesis (ECO) media, with and without plant growth regulators (PGRs). The embryos reached the cotyledonary stage in 9 weeks, but those cultured on ECO medium containing 0.1 mg·L^−1^ 6-(dimethylallylamino)purine (2iP), 0.1 mg·L^−1^ 6-benzyladenine (BA) and 0.05 mg·L^−1^ indole-3-butyric acid (IBA) exhibited the largest sizes, with an average of 4.7 mm. Somatic embryo conversion into plantlets was evaluated using different culture media (half-strength OM or one-third strength Murashige and Skoog (MS)), light conditions (light or dark) and desiccation pretreatments. The highest rate of somatic embryo conversion (45%) was observed under a 16 h photoperiod on half strength OM medium containing 0.1 mg·L^−1^ gibberellic acid (GA_3_) and 0.1 mg·L^−1^ 1-naphthalene acetic acid (NAA). The embryos that failed to germinate showed either necrosis, cotyledon greening with no further conversion, adventitious bud formation or secondary embryogenesis. The findings of this study will be beneficial for biotechnological applications in olive.

## 1. Introduction

Olive (*Olea europaea* L.) belongs to the *Oleaceae* family and is an economically important species native to the eastern Mediterranean, Asia Minor and northern Iraq, cultivated for fruits and edible oil production [1,2,3]. 

Olive propagation and genetic improvement are generally performed by conventional techniques. Olive is commercially propagated by stem cuttings. However, in many genotypes, vegetative propagation through cuttings is hampered due to recalcitrance to rooting [4]. Genetic improvement through conventional cross-breeding is limited by many factors including the long juvenile period, high heterozygosis and seed germination difficulties [5].

In vitro culture of olive could be a good alternative to conventional methods. In fact, tissue culture techniques such as somatic embryogenesis have been applied to several plant species, not only to produce a large number of healthy plants in a short period of time, but also for pathogen elimination, synthetic seed production, cryopreservation and genetic transformation purposes [6,7]. 

Somatic embryogenesis refers to the process by which bipolar structures, called somatic embryos, are developed from cells without fertilization [8]. Researches on somatic embryogenesis of olive started in the 1980s [9], and since then, divergent results were reported. In fact, many factors were found to affect somatic embryogenesis in olive. For example, explant type, basal formulation of culture medium, plant growth regulators (PGRs), carbon sources, medium texture and light conditions. Brhadda et al. [10,11] compared the effects of the basal formulation of culture medium, photoperiod and different concentrations of sucrose, mannitol and sorbitol on somatic embryogenesis from cotyledon explants of cv. Picholine Marocaine, and suggested to culture the proximal part of cotyledons on Murashige and Skoog (MS) medium supplemented with 30 g·L^−1^ sucrose under dark conditions. Trabelsi et al. [12] evaluated the effects of different induction periods, carbon sources, nitrogen sources and PGRs on somatic embryogenesis from cotyledon segments of three olive cultivars, Chetoui, Chemlali and Arbequina, and recommended a 4-week induction period on a medium containing 30–40 g·L^−1^ sucrose, low PGR concentrations and both organic and inorganic nitrogen forms. Capelo et al. [13] studied the influence of different basal formulations of culture medium, PGR combinations and explants on somatic embryogenesis of the wild olive tree (*Olea europaea* ssp. *europaea* var. *sylvestris*) and reported that distal blade leaf and petiole explants cultured on MS medium containing 12.25 μM indole-3-butyric acid (IBA) and 4.56 μM zeatin resulted in the highest somatic embryogenesis rates. 

Somatic embryogenesis of olive was mainly achieved from zygotic explants [14,15]. In fact, adult tissues are recalcitrant to somatic embryogenesis and consequently, very few studies reported successful regeneration from this type of explants [13,16]. Thus, in the recent years, efforts have been made to overcome the recalcitrant behavior of adult explants of elite olive cultivars [17,18,19].

In addition to the above-mentioned factors, it is well known that in vitro regeneration of olive through somatic embryogenesis is highly genotype-dependent [20]. Indeed, somatic embryogenesis has been successfully achieved for some olive cultivars and other biotechnological applications were performed. For example, in cvs. Picual and Canino, regeneration through somatic embryogenesis as well as genetic transformation and cryopreservation protocols were reported [21,22,23,24,25,26,27]. However, in some other olive cultivars as well as in the wild olive *Olea europaea* ssp. *europaea* var. *sylvestris*, somatic embryogenesis was induced but embryo conversion into plantlets was not reported [13,19,28]. In fact, somatic embryo germination, along with the recalcitrant behavior of adult explants, are the main bottlenecks that hamper the wide and routine use of this technique in olive [14]. Developing efficient regeneration systems through somatic embryogenesis for the elite olive cultivars will open new opportunities for the improvement of this species. 

Somatic embryo maturation is an important process that affects the subsequent germination ability of embryos [15]. Thereby, successful germination could be considered as a good indicator of the efficiency of the maturation treatment [29]. Benzekri and Sánchez-Romero [29] evaluated the effects of different factors on the maturation of somatic embryos of cv. Picual and suggested the use of abscisic acid (ABA)-free olive cyclic embryogenesis (ECO) medium supplemented with 20 g·L^−1^ sucrose. Cerezo et al. [22] reported that semi-permeable cellulose acetate membranes significantly enhanced the conversion frequency of somatic embryos of cv. Picual. Regarding somatic embryo germination and conversion, no standard medium or culture conditions were established [15,30]. In fact, different basal formulations, mostly consisting of variations of MS and olive medium (OM), were used [15]. Rugini and Caricato [16] suggested to culture mature embryos in agitated liquid medium for 6 days in the dark before transferring them to light conditions. Rugini [31] recommended the use of a low mineral salt medium supplemented with zeatin for embryo germination. On the other hand, many authors used PGR-free media for the germination of olive somatic embryos [15].

Dahbia cv. is an elite Moroccan olive cultivar characterized by a short juvenile period and high fruit quality [32]. Previous studies highlighted the significant influence of PGRs and explant type on somatic embryogenesis induction in this cultivar [17,20,33]. Indeed, somatic embryogenesis was observed from radicles and cotyledons excised from zygotic embryos as well as from leaf explants, but not from ovaries, stamens and petioles. Besides, medium texture, induction period and cytokinin type were all reported to influence somatic embryogenesis induction from leaf explants. In fact, only a short induction period of 4 days on liquid agitated medium containing thidiazuron (TDZ) resulted in somatic embryo induction. In addition, histological observations were performed to illustrate the morphogenetic pathway of this developmental process. However, somatic embryo germination was not achieved [17,20,33].

During somatic embryogenesis, morphogenetic responses such as secondary embryogenesis and organogenesis may occur [9,17,20], which can be used for different biotechnological applications. Besides, secondary embryogenesis allows the establishment of cycling cultures, thus enhancing the frequency of somatic embryo production in plants recalcitrant to somatic embryogenesis [34]. Accordingly, it is important to determine the specific conditions under which these different morphogenetic responses occur. 

The purpose of the present study was to evaluate the effect of different PGRs and culture conditions in order to develop an efficient and simple protocol for somatic embryo maturation and conversion from juvenile explants of olive cv. Dahbia. Besides, the different morphogenetic responses that may occur during this process were monitored.

## 2. Results

### 2.1. Somatic Embryo Maturation

The growth and development of globular somatic embryos were monitored every 3 weeks. The findings showed that somatic embryo growth varies depending on culture medium (Table 1). After 3 weeks of culture, the size of somatic embryos ranged from 1.5 to 2.1 mm. After 6 weeks of culture, the size of somatic embryos reached 3.8 mm. After 9 weeks of culture, the somatic embryos cultured on ECO medium supplemented with 0.1 mg·L^−1^ 6-(dimethylallylamino) purine (2iP), 0.1 mg·L^−1^ 6-benzyladenine (BA) and 0.05 mg·L^−1^ IBA exhibited the largest sizes, with an average of 4.7 mm (Table 1). Besides, the development of somatic embryos from the globular stage to the heart-shaped and cotyledonary stages was observed. In fact, after the first 3 weeks of culture, the embryos showed an increase in size and acquired a heart shape. However, 5% of the embryos cultured on PGR-free ECO medium, and 10% of those cultured on PGR-free half-strength OM medium, showed cotyledonary fusion and thus were discarded. During the next 6 weeks of culture, the somatic embryos kept increasing in size while progressing to the cotyledonary stage. During the last 3 weeks of culture, a slight browning was observed in the root apex of some somatic embryos. However, this browning did not affect their subsequent germination. At the end of the maturation period, all the embryos reached the cotyledonary stage regardless of the maturation medium. 

### 2.2. Somatic Embryo Conversion into Plantlets 

#### 2.2.1. Effects of PGRs 

**Figure 1:**
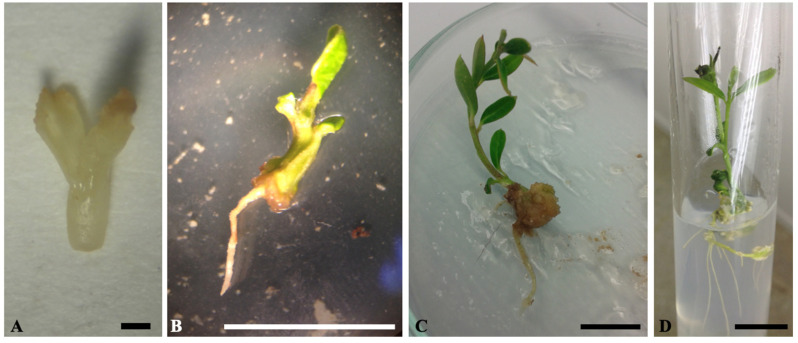
Somatic embryo germination and conversion into plantlet under 16 h photoperiod. (**A**) Mature somatic embryo. (**B**) Somatic embryo germination on half-strength OM medium supplemented with 0.1 mg·L^−1^ GA3 and 0.1 mg·L^−1^ NAA. (**C**) and (**D**) Somatic embryo development and conversion into complete plantlet. Bars correspond to 1 mm (A) and 1 cm (B–D).

**Figure 2:**
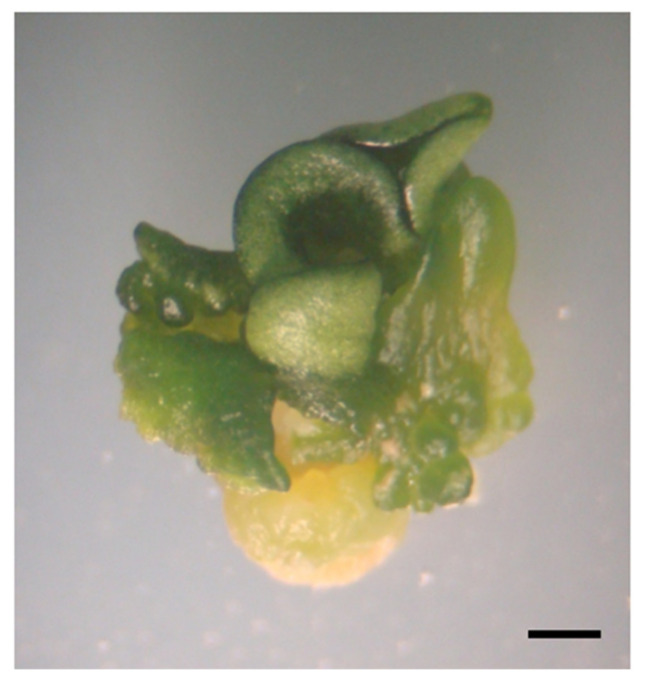
Somatic embryo germination on one-third strength MS medium containing 10 g L^−1^ sucrose, showing cotyledon greening with no root development. The bar corresponds to 1 mm.

The effects of different PGRs on somatic embryo germination and conversion into plantlets (Figure 1) were evaluated. The findings of this experiment showed that combining 0.1 mg L^−1^ gibberellic acid (GA_3_) with 0.1 mg·L^−1^ 1-naphthalene acetic acid (NAA) resulted in the highest germination and conversion rate (45%) (Table 2). This was followed by half-strength OM medium supplemented with 0.1 mg·L^−1^ NAA, which showed a germination rate of 40%. These germination rates were higher than that (5%) obtained on half-strength OM medium supplemented with 0.1 mg·L^−1^ GA_3_ (Table 2). On the other hand, no embryo germination was observed when used zeatin or no PGRs were added. When one-third strength MS medium containing 10 g·L^−1^ sucrose was used, 65% of somatic embryos showed cotyledon greening, but with no radicle emergence and consequently no conversion (Figure 2). 

Based on the findings of this experiment, half-strength OM medium supplemented with 0.1 mg·L^−1^ GA_3_ and 0.1 mg·L^−1^ NAA is recommended for the germination and conversion of somatic embryos derived from seeds of cv. Dahbia.

**Figure 3:**
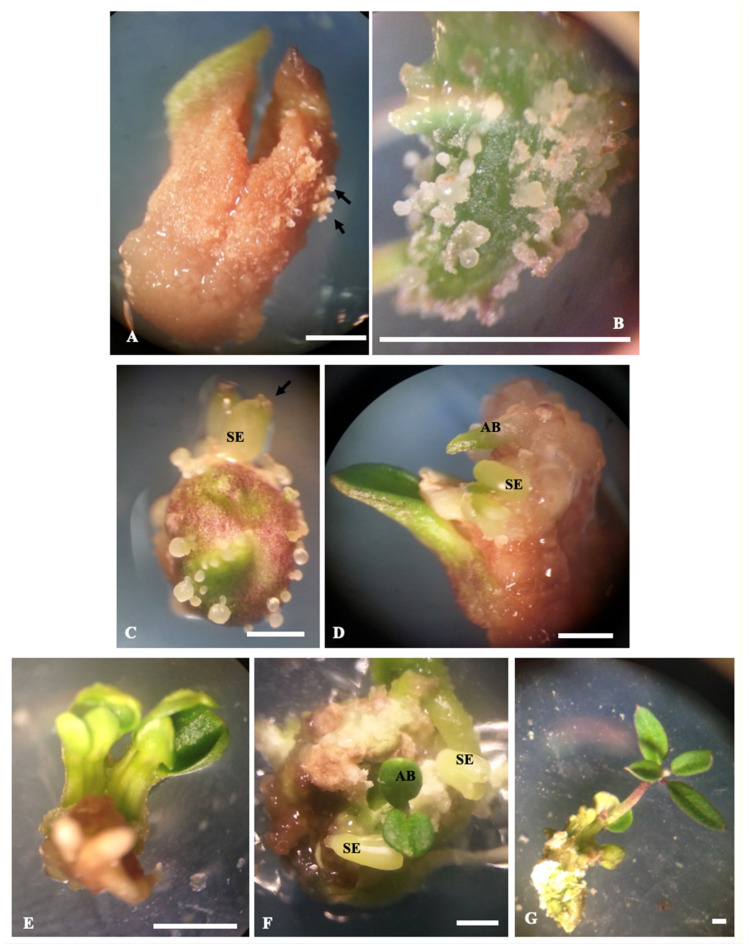
Secondary embryogenesis and adventitious bud formation during somatic embryo germination. (**A**) and (**B**) Beginning of development of globular secondary embryos (arrows) from the primary ones, (**C**) Secondary embryos at globular and heart-shaped (arrow) stages, (**D**) Beginning of development of an adventitious bud, (**E**) Adventitious buds, (**F**) Heart-shaped secondary embryos and adventitious buds, (**G**) Adventitious bud singled out from a primary somatic embryo by using a scalpel. AB—Adventitious bud. SE—somatic embryo. Bars correspond to 1 mm.

It is worth noting that, before germination, callus formation may occur in the radicle region of some somatic embryos. However, this callogenesis did not hamper somatic embryo germination. The findings of the present study also showed that, in many cases, somatic embryos fail to germinate, but instead show secondary somatic embryogenesis or adventitious bud formation (Figure 3). Adventitious buds and secondary embryos may be developed from any part (i.e., the radicle part and the cotyledonary part) of the mature primary embryo placed on germination medium, either directly or after callus formation. When adventitious bud formation occurred, never more than two buds are formed per primary somatic embryo. Adventitious buds keep growing attached to the somatic embryo. At a certain point, they should be separated from the embryo by using a scalpel and then transferred to a fresh culture medium for further growth and development. The frequency of adventitious bud formation and secondary embryogenesis depended on culture medium composition and culture conditions. In this experiment, the highest rate of secondary embryogenesis (30%) was observed on half-strength OM medium supplemented with 0.1 mg L^−1^ NAA, while the highest rate of adventitious bud formation (35%) occurred on half-strength OM medium supplemented with 0.1 mg·L^−1^ GA_3_ and 0.1 mg·L^−1^ NAA. Secondary embryos showed the same developmental pattern as the primary ones. They were first at the globular stage then developed into the heart-shaped stage. Besides, they could be easily detached from the mother tissue. On the other hand, adventitious buds arose as green leafy structures and were strongly connected to the mother tissue. In fact, it was necessary to use a scalpel to separate them.

#### 2.2.2. Effects of Light Conditions 

The results of this experiment showed that culturing mature somatic embryos in the dark results in a lower germination rate (35%) than that observed under the 16 h photoperiod. Moreover, embryo development was delayed under dark conditions. On the other hand, dark conditions enhanced the secondary embryogenesis rate (30%) and showed an adventitious bud formation rate of 5% (Table 3).

#### 2.2.3. Effects of Sucrose and Mannitol Pretreatments

In this experiment, mature somatic embryos were cultured for 4 weeks on media containing different concentrations of sucrose, alone or in combination with mannitol to induce desiccation before transferring them to the germination medium. According to Table 4, desiccation pretreatments did not improve somatic embryo germination. The highest germination rate (20%) was observed when mature embryos were cultured on half-strength OM medium supplemented with 40 g·L^−1^ sucrose, or with 20 g·L^−1^ sucrose and 20 g·L^−1^ mannitol, which was lower than that observed in the control (45%). The other pretreatments resulted in lower germination rates (10–15%). Besides, desiccation pretreatments resulted in secondary embryogenesis rates ranging from 10 to 25% and high adventitious bud formation rates of up to 50%. Based on the results of the present study, desiccation pretreatments do not improve the germination of somatic embryos derived from seeds of cv. Dahbia.

### 2.3. Plantlet Acclimatization 

At the end of the germination and conversion experiments, the plantlets obtained under the optimal conditions (i.e., half-strength OM medium + 0.1 mg·L^−1^ GA_3_ + 0.1 mg·L^−1^ NAA + 30 g·L^−1^ sucrose, 16 h photoperiod, no pretreatment) were transferred to the glasshouse, where they all survived and exhibited normal growth and development.

## 3. Discussion

To date, maturation, germination and conversion of olive somatic embryos have been scarcely studied. In fact, regeneration through somatic embryogenesis is difficult to achieve in olive, and the results reported in literature concern only a limited number of cultivars. However, it is well known that olive response to in vitro manipulations is highly genotype-dependent [20,35]. Besides, many studies have only reported the induction of somatic embryogenesis, but did not report subsequent maturation and conversion of somatic embryos [15]. Therefore, it seems important to carefully optimize the culture conditions in order to successfully develop efficient regeneration systems for the best olive cultivars.

In order to develop a rapid, simple and efficient protocol for somatic embryo maturation and conversion, the effects of different PGRs and culture conditions were evaluated. Somatic embryo maturation was carried out on two basal formulations, half-strength OM and ECO, with and without PGRs. It was found that the embryos cultured on ECO medium supplemented with 0.1 mg·L^−1^ 2iP, 0.1 mg·L^−1^ BA and 0.05 mg·L^−1^ IBA exhibited the largest sizes. Cerezo et al. [22] evaluated the effect of two basal formulations (OMc and ECO) without PGRs and supplemented with 1 g·L^−1^ activated charcoal, as well as that of semi-permeable cellulose acetate membranes on somatic embryo maturation of cv. Picual, and suggested the use of ECO medium and semi-permeable cellulose acetate membranes as they significantly improve subsequent germination of somatic embryos. In a previous study on cvs. Arbequina and Dahbia, somatic embryo maturation was carried out on ECO medium containing 1 g·L^−1^ activated charcoal for 9 weeks, and the average sizes of mature embryos were 2.8 and 3.7 mm in Dahbia and Arbequina, respectively [20]. The findings of the present study showed that adding PGRs to ECO medium resulted in larger sizes of somatic embryos, with an average of 4.7 mm in cv. Dahbia. Besides, a rapid development of somatic embryos from the globular to cotyledonary stages was observed. The promoting effect of PGRs on somatic embryo maturation was observed in other plant species such as kodo millet (*Paspalum scorbiculatum* Linn.) and half-high blueberry (*Vaccinium corymbosum* L. × *V. angustifolium*) [36,37]. Indeed, PGRs are known to stimulate plant growth and development in vitro as they interact with the endogenous plat hormones. This interaction results in cell division, differentiation and growth [38,39,40]. 

For somatic embryo germination and conversion, the effects of PGRs, photoperiod and different pretreatments were investigated. After 3 months of culture, up to 45% of somatic embryos of cv. Dahbia successfully germinated. The highest germination rate was observed under the 16 h photoperiod on half-strength OM medium containing 0.1 mg·L^−1^ GA_3_ and 0.1 mg·L^−1^ NAA. The promotive effect of GA_3_ and NAA on somatic embryo germination was reported in many plant species such as *Oplopanax elatus* [41], *Panax ginseng* [42], *Mondia whitei* [43] and *Phoenix dactylifera* [44]. In all these studies, NAA and GA_3_ were added alone to the germination medium. In the present study, the combination of these two PGRs showed the highest germination frequency and thus is recommended for somatic embryo germination of olive cv. Dahbia. The results of the present investigation showed that one-third strength MS medium supplemented with 10 g·L^−1^ sucrose did not promote somatic embryo germination. In fact, the use of this medium showed cotyledon greening and enlargement but with no root formation. One-third strength MS medium supplemented with 10 g·L^−1^ sucrose was recommended for cv. Picual and genotype StopVert [22,35]. In a recent study on olive cv. Galega Vulgar, somatic embryo conversion was performed on PGR-free OMc medium [45]. The findings of the present study along with those from the literature highlighted clearly the specific requirement of each olive genotype for somatic embryo germination and subsequent development. 

The results of this investigation showed that maintaining embryos under a 16 h photoperiod resulted in a higher somatic embryo germination rate than in dark condition. Afreen et al. [46] reported that culturing somatic embryos under light conditions promotes the development of photosynthetic pigments and functional stomata, increases the chlorophyll level and enhances the photosynthetic ability. On the other hand, desiccation pretreatments did not improve somatic embryo germination. In fact, mature somatic embryos were cultured for four weeks on PGR-free half-strength OM medium supplemented with different concentrations of sucrose and mannitol to induce desiccation. According to Kermode et al. [47], desiccation reduces the sensitivity of embryos to ABA and thus may promote somatic embryo germination and subsequent development. The results of the present study showed that desiccation pretreatments do not improve somatic embryo germination in olive cv. Dahbia. Similar results were also observed in date palm [44]. 

During the germination process, secondary embryogenesis and adventitious bud formation were also observed. Secondary embryogenesis is the process by which new somatic embryos are formed on the surface of the primary ones. This phenomenon is highly interesting as it allows efficient somatic embryo production, and thus various biotechnological applications of somatic embryogenesis (e.g., large-scale propagation, genetic transformation and cryopreservation). Olive secondary embryogenesis was also reported by Rugini [9] and Pires et al. [45]. Secondary embryogenesis can be used to establish cycling cultures as it provides a long-term source of somatic embryos, and thus significantly enhances the frequency of somatic embryo production. This is highly valuable for both fundamental studies and further applications of somatic embryogenesis in olive. Regarding adventitious shoot bud formation, very few studies reported the occurrence of this morphogenesis in olive. Cañas and Benbadis [48] established a regeneration system through indirect organogenesis (adventitious shoot and root induction) in olive cvs. Tanche and Picual by using cotyledon-derived calli. Mencuccini and Rugini [49] reported shoot formation from petiole explants of olive in the presence and absence of callus, with up to 18.7% shoot regeneration rate in cv. Moraiolo. Mazri et al. [17] reported adventitious bud formation from petiole explants of cv. Dahbia. In the present study, a high rate of adventitious bud formation (up to 50%) was observed. Adventitious shoot bud formation also represents a powerful tool that has been widely used for mass propagation of plants [50,51,52]. This regeneration system has been scarcely studied in olive. The findings of the present study open new perspectives in the field of olive micropropagation and genetic improvement.

## 4. Materials and Methods 

### 4.1. Plant Material and Establishment of Embryogenic Cultures

Embryogenic cultures (i.e., calli with globular somatic embryos) derived from seeds of olive (*Olea europaea* L.) cv. Dahbia were used in the present study. The embryogenic cultures were obtained from radicle explants according to the protocol described by Mazri et al. [20]. Briefly, seeds were taken from freshly harvested mature fruits and surface-sterilized with a solution of 10% commercial bleach for 10 min, followed by three rinses with sterile distilled water. The seeds were kept in sterile distilled water for 48 h in darkness, then a second surface-sterilization was applied. Afterwards, zygotic embryos were excised and radicle explants were cultured on OMc medium [48] supplemented with 25 μM IBA and 2.5 μM 2iP for 3 weeks. The explants were then transferred to PGR-free OMc medium for 4 weeks, and finally to ECO medium, which consists of ¼OM macro-elements [53], ¼MS medium microelements [54], ½OM vitamins, 50 mg·L^−1^ myo-inositol, 550 mg L^−1^ l-glutamine, 20 g·L^−1^ sucrose [22], and supplemented with 3 g·L^−1^ phytagel, 0.1 mg·L^−1^ 2iP, 0.1 mg·L^−1^ BA and 0.05 mg·L^−1^ IBA. The explants were kept under dark conditions at 25 °C. The embryogenic cultures were maintained in proliferation for one year on ECO medium supplemented with 3 g·L^−1^ phytagel, 0.1 mg·L^−1^ 2iP, 0.1 mg·L^−1^ BA and 0.05 mg·L^−1^ IBA, with monthly subcultures onto fresh medium. All chemicals were purchased from Sigma-Aldrich (Steinheim, Germany).

### 4.2. Somatic Embryo Maturation 

Globular somatic embryos were cultured on ECO or half-strength OM basal formulations, without PGRs or supplemented with 0.1 mg·L^−1^ 2iP, 0.1 mg·L^−1^ BA and 0.05 mg·L^−1^ IBA. All media were solidified with 3 g·L^−1^ phytagel and their pH was adjusted to 5.74 with 0.1 N KOH and/or 0.1 N HCl before autoclaving at 121 °C for 20 min. The cultures were maintained under dark conditions at 25 °C for 9 weeks with subculturing to fresh medium at 3-week intervals. During this experiment, 4 globular somatic embryos were placed per Petri dish (10 cm diameter), which was considered as one replication, and for each treatment, 10 Petri dishes were used. The size of somatic embryos was measured every 3 weeks.

### 4.3. Somatic Embryo Conversion into Plantlets

#### 4.3.1. Effects of PGRs 

Before starting germination experiments, globular somatic embryos were first cultured on ECO medium supplemented with 0.1 mg·L^−1^ 2iP, 0.1 mg·L^−1^ BA and 0.05 mg·L^−1^ IBA for 9 weeks to reach maturity. Somatic embryos at the cotyledonary stage and larger than 4 mm (i.e., mature somatic embryos) were used in the germination experiments. Two embryos were placed per Petri dish containing half-strength OM medium supplemented with 30 g·L^-1^ sucrose and different PGRs: GA_3_ (0.1 mg·L^−1^) and NAA (0.1 mg·L^−1^) either alone or in combination, or zeatin (0.5-1 mg·L^−1^). Besides, two control media were used: PGR-free half-strength OM medium supplemented with 30 g·L^−1^ sucrose and one-third strength MS medium containing 10 g·L^−1^ sucrose, which was suggested for cv. Picual [22]. All culture media were solidified with 8 g·L^−1^ agar and their pH was adjusted to 5.74 before autoclaving at 121 °C for 20 min. The cultures were maintained at a 16 h photoperiod and 25°C, and were transferred to fresh medium at 4-week intervals. During this experiment, 10 Petri dishes were used per treatment.

#### 4.3.2. Effects of Light Conditions 

Based on the findings of the first experiment, mature somatic embryos were cultured on half-strength OM medium supplemented with 0.1 mg·L^−1^ GA_3_, 0.1 mg·L^−1^ NAA, 8 g·L^−1^ agar and 30 g·L^−1^ sucrose. The cultures were kept either under a 16 h photoperiod or in darkness at 25 °C. Two mature somatic embryos were placed per Petri dish and 10 Petri dishes were used per treatment.

#### 4.3.3. Effects of Sucrose and Mannitol Pretreatments 

The effects of different pretreatments on somatic embryo germination were evaluated. Mature somatic embryos were cultured on PGR-free half-strength OM medium supplemented with different concentrations of sucrose (30–60 g·L^−1^), or with combinations of sucrose and mannitol (15–30 g·L^−1^ each) for 4 weeks under dark conditions at 25 °C. Afterwards, the somatic embryos were transferred to half-strength OM medium supplemented with 0.1 mg·L^−1^ GA_3_, 0.1 mg·L^−1^ NAA and 30 g·L^−1^ sucrose under a 16 h photoperiod. All culture media were solidified with 8 g·L^−1^ agar. Two mature somatic embryos were placed per Petri dish and 10 Petri dishes were used per treatment. The results of this experiment were compared to those in which mature somatic embryos were transferred directly from maturation medium to germination medium, with no pretreatment, which was considered as a control.

### 4.4. Plantlet Acclimatization

At the end of the germination and conversion experiments, the plantlets obtained under the optimal conditions were transferred to the glasshouse for subsequent growth and development. Briefly, the plantlets were removed from culture vessels and their root system was gently rinsed with running water to get rid of adhering medium. The plantlets were then placed in plastic pots filled with a 1:1 peat-sand mixture (*w/w*), covered with a transparent glass cover and kept in the culture room for 30 days (16 h photoperiod, 25 °C). Afterwards, the glass covers were removed and the plantlets were transferred to the glasshouse and covered with a plastic bag for 15 days to maintain high relative humidity (RH). The plastic bag was then removed progressively over a period of 15 days to acclimatize the plantlets to glasshouse conditions (27 °C, 70% RH).

### 4.5. Data Collection 

After 3 months of culture on germination media, the germination and conversion percentage of somatic embryos, the percentage of secondary embryogenesis, the percentage of adventitious bud formation, the percentage of embryos showing cotyledon greening and enlargement, the percentage of necrotic embryos and the morphological aspect of the germinated somatic embryos were recorded. 

### 4.6. Statistical Analysis 

All experiments were conducted as a completely randomized design. Maturation data were subjected to analysis of variance. Mean values were separated by the Student-Newman-Keuls (SNK) test at the 5% significance level. The frequencies of somatic embryo conversion, secondary embryogenesis, adventitious bud formation and necrosis influenced by different culture media and conditions were compared using a Chi-square analysis at 5% level of significance. All statistical calculations were made using SPSS (v. 26, IBM, Chicago, IL, USA). 

## 5. Conclusions

A simple protocol for rapid and efficient maturation and conversion of somatic embryos derived from seeds of olive cv. Dahbia was reported. Maturation of globular somatic embryos was performed on ECO medium supplemented with 0.1 mg·L^−1^ 2iP, 0.1 mg·L^−1^ BA and 0.05 mg·L^−1^ IBA. Somatic embryo germination and conversion were achieved on half-strength OM medium supplemented with 0.1 mg·L^−1^ GA_3_ and 0.1 mg·L^−1^ NAA. Besides, adventitious bud formation and secondary embryogenesis were observed. Hence, further studies should be carried out regarding secondary embryogenesis and adventitious bud formation in olive to explore the mechanisms responsible for their occurrence. In fact, these two morphogenetic responses have been scarcely studied in this species while they can have great potential in various biotechnological applications. The reported protocol will be further tested on somatic embryos derived from leaf explants of cv. Dahbia for clonal propagation purposes, as well as on other elite olive cultivars.

## Figures and Tables

**Table 1:** Effect of different culture media on somatic embryo maturation.

Culture Medium	Size (mm) of Somatic Embryos after 3, 6 and 9 Weeks of Culture		
	3 Weeks	6 Weeks	9 Weeks
ECO + PGRs	2.1 ± 0.3 ^b^	3.8 ± 1.3 ^b^	4.7 ± 0.8 ^b^
PGR-free ECO	1.6 ± 0.5 ^a^	2.2 ± 0.4 ^a^	3.0 ± 0.6 ^a^
Half-strength OM + PGRs	1.8 ± 0.4 ^ab^	3.1 ± 0.7 ^b^	4.2 ± 0.7 ^b^
PGR-free half-strength OM	1.5 ± 0.5 ^a^	1.9 ± 0.3 ^a^	2.8 ± 0.4 ^a^

Data are means ± standard deviation. Data in the same column followed by different letters are significantly different. ECO, olive cyclic embryogenesis medium; OM, olive medium; PGR, plant growth regulator: 0.1 mg L^−1^ 6-(dimethylallylamino) purine + 0.1 mg L^−1^ μM 6-benzyladenine + 0.05 mg L^−1^ μM indole-3-butyric acid.

**Table 2:** Effect of different culture media on somatic embryo germination and conversion into plantlets.

Culture Medium	Germination and Conversion into Plantlets (%)	Secondary Embryogenesis (%)	Adventitious Bud Formation (%)	Necrosis (%)
Half-strength OM + 0.1 mg·L^−1^ GA_3_ + 30 g·L^−1^ sucrose	5 ^ab^	20 ^ab^	15 ^ab^	60 ^bc^
Half-strength OM + 0.1 mg·L^−1^ NAA + 30 g·L^−1^ sucrose	40 ^bc^	30 ^b^	5 ^ab^	25 ^ab^
Half-strength OM + 0.1 mg·L^−1^ GA_3_ + 0.1 mg·L^−1^ NAA + 30 g·L^−1^ sucrose	45 ^c^	15 ^ab^	35 ^b^	5 ^a^
Half-strength OM + 0.5 mg·L^−1^ zeatin + 30 g·L^−1^ sucrose	0 ^a^	0 ^a^	15 ^ab^	85 ^c^
Half-strength OM + 1 mg·L^−1^ zeatin + 30 g·L^−1^ sucrose	0 ^a^	0 ^a^	0 ^a^	100 ^c^
Half-strength OM + 30 g·L^−1^ sucrose	0 ^a^	0 ^a^	0 ^a^	100 ^c^
One-third strength MS + 10 g·L^−1^ sucrose	0 ^a^	0 ^a^	0 ^a^	35 ^ab^

Means in the same column followed by different letters are significantly different. GA_3_—gibberellic acid; MS—Murashige and Skoog medium; NAA—1-naphthalene acetic acid; OM—olive medium.

**Table 3:** Effect of light conditions on somatic embryo germination and conversion into plantlets.

Light Conditions	Germination and Conversion into Plantlets (%)	Secondary Embryogenesis (%)	Adventitious Bud Formation (%)	Necrosis (%)
16 h photoperiod	45 ^a^	15 ^a^	35 ^a^	5 ^a^
Dark conditions	35 ^a^	30 ^a^	5 ^a^	30 ^a^

Means in the same column followed by different letters are significantly different.

**Table 4:** Effect of different pretreatments on somatic embryo germination and conversion into plantlets.

Pretreatment Medium	Germination and Conversion into Plantlets (%)	Secondary Embryogenesis (%)	Adventitious Bud Formation (%)	Necrosis (%)
No pretreatment	45 ^b^	15 ^a^	35 ^ab^	5 ^a^
Half-strength OM + 30 g·L^−1^ sucrose	15 ^ab^	20 ^a^	50 ^b^	15 ^a^
Half-strength OM + 40 g·L^−1^ sucrose	20 ^ab^	20 ^a^	50 ^b^	10 ^a^
Half-strength OM + 50 g·L^−1^ sucrose	15 ^ab^	20 ^a^	50 ^b^	15 ^a^
Half-strength OM + 60 g·L^−1^ sucrose	10 ^a^	20 ^a^	45 ^b^	25 ^a^
Half-strength OM + 15 g·L^−1^ sucrose + 15 g·L^−1^ mannitol	15 ^ab^	20 ^a^	15 ^a^	50 ^b^
Half-strength OM + 20 g·L^−1^ sucrose + 20 g·L^−1^ mannitol	20 ^ab^	25 ^a^	20 ^a^	35 ^ab^
Half-strength OM + 25 g·L^−1^ sucrose + 25 g·L^−1^ mannitol	15 ^ab^	15 ^a^	20 ^a^	50 ^b^
Half-strength OM + 30 g·L^−1^ sucrose + 30 g·L^−1^ mannitol	15 ^ab^	10 ^a^	20 ^a^	55 ^b^

Means in the same column followed by different letters are significantly different. OM—olive medium.
